# The role of probiotic supplementation in reducing *Helicobacter pylori* recurrence after classic quadruple therapy

**DOI:** 10.3389/fphar.2025.1621090

**Published:** 2025-07-14

**Authors:** Jie Zhao, Jieyun Chen, Yanqing Wang, Chen Zhu, Chenjing Xia, Wei Yang

**Affiliations:** ^1^ Department of Gastroenterology, Jinxi People’s Hospital of Kunshan, (Kunshan Elderly Hospital), Kunshan, China; ^2^ Department of Geriatrics, Jinxi People’s Hospital of Kunshan, (Kunshan Elderly Hospital), Kunshan, China; ^3^ Department of Gastroenterology, Kunshan Hospital of Traditional Chinese Medicine, Kunshan Affiliated Hospital of Nanjing University of Chinese Medicine, Kunshan, China

**Keywords:** *Helicobacter pylori*, probiotic supplementation, quadruple therapy, recurrence, gut microbiota

## Abstract

**Background:**

*Helicobacter pylori* (*H. pylori*) infection remains a global public health issue, closely linked to peptic ulcer disease, gastric cancer, and MALT lymphoma. Although classic bismuth-containing quadruple therapy achieves high eradication rates, recurrence after treatment is still a significant concern. Recent evidence suggests that probiotics may support gastrointestinal homeostasis, modulate immune responses, and help prevent *H. pylori* reinfection. This study aimed to evaluate whether probiotic supplementation reduces *H. pylori* recurrence following classic quadruple therapy.

**Method:**

In this retrospective cohort study, we analyzed data from 305 adult patients with confirmed *H. pylori* infection treated at Kunshan Hospital of Traditional Chinese Medicine and Jinxi People’s Hospital of Kunshan between January 2023 and December 2023. All patients received standard quadruple therapy for 14 days. Among them, 127 patients additionally received probiotic supplementation during and for 4 weeks following eradication therapy, while 178 did not. Propensity score matching (1:1) was performed to balance baseline covariates, including age, sex, BMI, smoking status, dietary habits, and prior *H. pylori* infection history. The primary outcome was *H. pylori* recurrence within 12 months post-treatment, confirmed by 13C-urea breath test. Secondary outcomes included treatment-related gastrointestinal symptoms, medication adherence, and adverse events.

**Results:**

After PSM, 120 matched pairs were analyzed. The recurrence rate of *H. pylori* was significantly lower in the probiotic group (9.2%) compared to the control group (19.2%) (P = 0.021). Patients in the probiotic group also reported a lower incidence of gastrointestinal discomfort, including bloating and antibiotic-associated diarrhea (P < 0.05), and demonstrated higher treatment adherence (91.7% vs. 83.3%, P = 0.038). No serious adverse events were reported in either group.

**Conclusion:**

Probiotic supplementation is associated with a reduced risk of *H. pylori* recurrence after classic quadruple therapy. In addition to improving gastrointestinal tolerance and adherence, probiotics may contribute to maintaining gut microbial balance and enhancing eradication durability. These findings support the integration of probiotics as an adjunct to standard therapy. Further randomized controlled trials are needed to confirm efficacy and identify optimal probiotic formulations and duration.

## Introduction

*Helicobacter pylori* (*H. pylori*) is a spiral-shaped Gram-negative bacterium that colonizes the gastric mucosa and affects more than 50% of the global population (Burucoa and Axon, 2017; Camilo et al., 2017; Duan et al., 2025). It is strongly associated with a wide range of gastrointestinal diseases, including chronic gastritis, peptic ulcer disease, mucosa-associated lymphoid tissue (MALT) lymphoma, and gastric cancer (Duan et al., 2025; Maleki Kakelar et al., 2019; Mentis et al., 2019). Despite the widespread use of bismuth-containing quadruple therapy as a first-line eradication regimen, recurrence following treatment remains a substantial clinical challenge, particularly in areas with high reinfection rates and increasing antibiotic resistance (Ding et al., 2023; Zhou et al., 2024).

The recurrence of *H. pylori* infection may result from either true recrudescence or reinfection and is influenced by host immunity, environmental factors, and disruption of the gut microbial ecosystem caused by broad-spectrum antibiotics (Xue et al., 2019). These microbiota disturbances can impair mucosal defenses, increase gastric pH, and reduce colonization resistance, thus creating a favorable niche for *H. pylori* to reestablish (Xu et al., 2024). In recent years, probiotics—live microorganisms that confer health benefits when administered in adequate amounts—have gained attention for their role in restoring intestinal microbial balance, modulating host immunity, and reducing gastrointestinal side effects during and after antibiotic therapy (Xu et al., 2024).

Several randomized controlled trials and meta-analyses have suggested that probiotic supplementation may enhance eradication rates, improve patient tolerance, and reduce treatment-related adverse effects such as bloating and diarrhea (Gupta et al., 2024; Kim et al., 2021; Samara et al., 2022; Aragona et al., 2024). However, most studies have focused on the role of probiotics during eradication therapy, with limited data available on their potential in preventing recurrence after successful treatment. Moreover, heterogeneity in probiotic strains, dosage, treatment duration, and study designs has led to inconclusive evidence regarding long-term benefits.

To address this knowledge gap, the present study investigates the real-world effectiveness of probiotic supplementation in reducing *H. pylori* recurrence following classic quadruple therapy. Using a retrospective cohort design with propensity score matching (PSM) to minimize baseline bias, we compared recurrence rates and secondary outcomes—including gastrointestinal symptoms and treatment adherence—between patients who received probiotics and those who did not. We hypothesize that probiotic use may not only reduce recurrence risk but also contribute to better treatment tolerance and post-treatment gut health. By clarifying the role of probiotics in recurrence prevention, this study aims to support the development of evidence-based strategies for *H. pylori* management in clinical practice.

## Materials and methods

### Study design and patient selection

This retrospective cohort study was conducted at the Department of Gastroenterology of Kunshan Hospital of Traditional Chinese Medicine and Jinxi People’s Hospital of Kunshan to evaluate the effect of probiotic supplementation on the recurrence of *Helicobacter pylori* (*H. pylori*) infection after classic bismuth-based quadruple therapy. Patients were eligible for inclusion if they were 18 years or older, had confirmed *H. pylori* infection by urea breath test (UBT), and completed a 14-day course of quadruple therapy between January 2023 and December 2023. Exclusion criteria included incomplete clinical or follow-up records, known immunodeficiency, prior gastric surgery, concurrent probiotic use outside of protocol, or death during follow-up.

Of the 305 eligible patients, 127 received probiotic supplementation (probiotic group), while 178 did not (control group). Probiotic agents were administered alongside eradication therapy and continued for an additional 4 weeks. To reduce bias and improve comparability, propensity score matching (PSM) was conducted at a 1:1 ratio, resulting in 120 matched pairs (n = 240) for final analysis.

## Probiotic supplementation protocol

Patients in the probiotic group received oral probiotic supplements containing viable strains of *Lactobacillus* spp. and Bifidobacterium spp., at a minimum dose of 1 × 10^9^ CFU/day. Supplementation began concurrently with quadruple therapy and continued for 4 weeks following treatment completion. Patients were instructed to take probiotics at least 2 h apart from antibiotics to minimize antimicrobial interference. Adherence was monitored through follow-up calls and medication return.

The control group received standard quadruple therapy without probiotic support. Both groups were treated using a 14-day bismuth-containing quadruple regimen composed of a proton pump inhibitor (PPI), bismuth potassium citrate, tetracycline, and metronidazole, administered as per national guidelines.

### Propensity score matching (PSM)

To minimize selection bias, PSM was performed using logistic regression to generate propensity scores, with probiotic use as the dependent variable. Covariates included in the model were age, sex, body mass index (BMI), smoking status, alcohol use, prior *H. pylori* treatment, family history of gastric disease, baseline gastrointestinal symptoms, and dietary factors (e.g., spicy or pickled food consumption). Nearest-neighbor matching without replacement was applied using a caliper width of 0.2 of the standard deviation of the logit of the propensity score. Balance was evaluated using standardized mean differences (SMD), with SMD <0.1 considered indicative of good matching.

### Outcome measures and data collection

The primary outcome was *H. pylori* recurrence within 12 months after treatment, confirmed by 13C-UBT. Recurrence was defined as a positive UBT following an initially negative result post-eradication.

Secondary outcomes included gastrointestinal side effects (e.g., bloating, nausea, diarrhea), medication adherence (defined as completion of >90% prescribed therapy), and self-reported treatment tolerance.

Clinical data were extracted from electronic medical records and telephone follow-ups. Adverse events were recorded and classified as mild, moderate, or severe according to the Common Terminology Criteria for Adverse Events (CTCAE v5.0).

### Statistical analysis

Descriptive statistics were used to summarize patient demographics and clinical characteristics. Categorical variables were compared using the chi-square test or Fisher’s exact test, and continuous variables using the independent-samples t-test or Mann–Whitney U test, as appropriate. Binary logistic regression was employed to identify independent predictors of *H. pylori* recurrence. Receiver operating characteristic (ROC) curves were generated to evaluate the predictive accuracy of probiotic supplementation for key outcomes. All statistical analyses were performed using SPSS version 25.0 (IBM Corp., Armonk, NY, United States) and R software version 4.0.5. A two-tailed P value <0.05 was considered statistically significant. Sample size estimation was performed using PASS version 11.0, with an alpha of 0.05% and 80% power to detect a 10% difference in recurrence rate.

All patients were consecutively enrolled from the participating hospitals during the defined study period, ensuring an unbiased representation of routine clinical practice. To determine an adequate sample size, a power analysis was conducted using PASS software (version 11.0), based on a two-sided alpha of 0.05% and 80% power to detect a 10% absolute difference in *H. pylori* recurrence rates (i.e., 20% in control vs. 10% in probiotic group), as supported by prior literature and pilot observations. A chi-square test was used for the group comparison. Given that over 95% of patients completed follow-up, dropout adjustment was not applied in the calculation.

## Results

### Baseline characteristics of patients with and without probiotic supplementation

**FIGURE 1:**
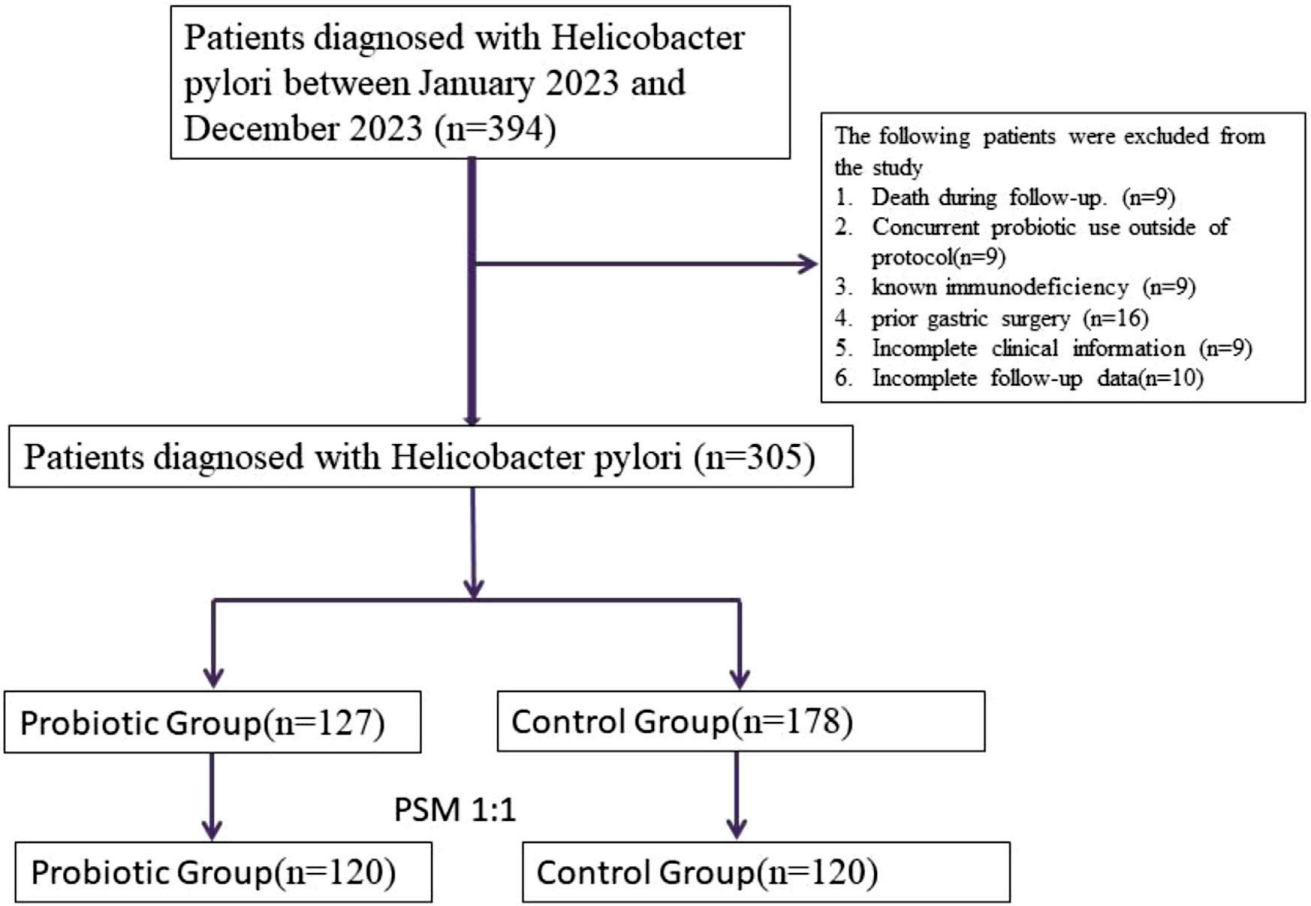
Flowchart of patient selection, exclusion, and 1:1 propensity score matching process in the *Helicobacter pylori* eradication cohort.

The selection, exclusion, and matching process is shown in Figure 1 to ensure transparent representation of patient flow throughout the study. A total of 305 patients diagnosed with *Helicobacter pylori* (*H. pylori*) infection who received standard quadruple therapy were enrolled in this study. Of these, 127 patients received additional probiotic supplementation (probiotic group), while 178 received standard treatment without probiotics (control group). After 1:1 propensity score matching based on age, sex, body mass index (BMI), lifestyle factors (smoking, alcohol use), prior *H. pylori* treatment history, gastrointestinal symptom burden, and dietary habits, a total of 120 matched pairs (n = 240) were included in the final analysis.

Before matching, patients in the probiotic group were slightly younger, had a higher proportion of female patients, and fewer prior treatment failures. After matching, no statistically significant differences were found between the two groups in demographic or clinical characteristics, including age, sex, BMI, smoking and drinking status, gastrointestinal symptom prevalence, dietary intake patterns (e.g., spicy or pickled food), or baseline *H. pylori* load. This balance in baseline features ensured comparability of outcomes (Table 1). An exploratory subgroup analysis comparing patients who received probiotic Brand A versus Brand B revealed no significant differences in recurrence rate, gastrointestinal adverse effects, or treatment adherence. These findings are presented in Supplementary Table S1.

**TABLE 1:** Baseline characteristics of patients with and without probiotic supplementation after propensity score matching (n = 240).

Variable	Probiotic group (n = 120)	Control group (n = 120)	P-value^a^
Age, years	43 (37–51)	44 (38–52)	0.472
Sex, male	66 (55.0%)	64 (53.3%)	0.784
BMI (kg/m^2^)	22.4 ± 2.9	22.6 ± 3.1	0.623
Smoking history	38 (31.7%)	36 (30.0%)	0.779
Alcohol consumption	29 (24.2%)	31 (25.8%)	0.772
Prior *H. pylori* treatment failure	15 (12.5%)	16 (13.3%)	0.849
Family history of gastric cancer	8 (6.7%)	9 (7.5%)	0.801
Chronic gastritis	96 (80.0%)	92 (76.7%)	0.541
Peptic ulcer history	32 (26.7%)	36 (30.0%)	0.589
Gastrointestinal symptoms (any)	51 (42.5%)	53 (44.2%)	0.781
Bloating	24 (20.0%)	26 (21.7%)	0.736
Diarrhea	9 (7.5%)	11 (9.2%)	0.641
Spicy food intake (frequent)	46 (38.3%)	49 (40.8%)	0.708
Pickled food intake (≥3x/week)	28 (23.3%)	30 (25.0%)	0.759
Probiotic brand A/B (%)	58/62	—	—
Treatment adherence (≥90%)	110 (91.7%)	100 (83.3%)	0.038^b^
Baseline UBT delta (‰)	19.2 ± 4.6	18.9 ± 4.8	0.651
Duration of therapy (days)	14 (standardized)	14	—
Adverse effect during treatment	15 (12.5%)	32 (26.7%)	0.006^b^
Antibiotic type (standard quadruple)	Yes	Yes	—

Values are presented as mean ± SD, median (IQR), or n (%) unless otherwise stated.

^a^ P-values calculated using Student’s t-test, Mann–Whitney U test, or χ2 test as appropriate.

^b^ Statistically significant (P < 0.05).

UBT: urea breath test; BMI: body mass index.

## Recurrence and clinical outcomes between groups

The primary outcome of this study was recurrence of *H. pylori* infection within 12 months after eradication therapy. The recurrence rate in the probiotic group was significantly lower than that in the control group (9.2% vs. 19.2%, P = 0.021). These findings suggest that adjunctive probiotic supplementation may contribute to more sustained bacterial clearance after standard therapy.

In terms of treatment tolerability, patients in the probiotic group experienced significantly fewer self-reported gastrointestinal adverse effects during and after treatment. The incidence of bloating was 12.5% in the probiotic group compared to 22.5% in the control group (P = 0.041), while antibiotic-associated diarrhea was reported in 7.5% versus 18.3% of patients, respectively (P = 0.013). Other side effects, such as nausea, abdominal pain, and taste disturbance, were reported with similar frequencies between groups and did not reach statistical significance.

Importantly, patients receiving probiotics exhibited significantly better treatment adherence, with 91.7% of patients completing ≥90% of their prescribed medications, compared to 83.3% in the control group (P = 0.038). Adherence is a critical determinant of eradication success, and this finding underscores the practical benefit of probiotic use in real-world settings. No serious adverse events were reported in either group. These outcome data are summarized in Table 2.

**TABLE 2:** Comparison of clinical outcomes between probiotic group and control group after matching (n = 240).

Variable	Probiotic group (n = 120)	Control group (n = 120)	P-value^a^
*H. pylori* recurrence (within 12 months)	11 (9.2%)	23 (19.2%)	0.021
Gastrointestinal adverse effects (any)	18 (15.0%)	36 (30.0%)	0.006
Bloating	12 (10.0%)	24 (20.0%)	0.041
Antibiotic-associated diarrhea	6 (5.0%)	22 (18.3%)	0.003
Nausea	8 (6.7%)	11 (9.2%)	0.478
Medication adherence (≥90%)	110 (91.7%)	100 (83.3%)	0.038
Incomplete therapy (<90%)	10 (8.3%)	20 (16.7%)	0.038
Patient-reported tolerance (score 1–10)	8.6 ± 1.1	7.4 ± 1.5	<0.001
Time to symptom relief (days)	4.3 ± 1.7	5.1 ± 2.1	0.014
30-day readmission for dyspepsia	2 (1.7%)	7 (5.8%)	0.088

Values are presented as n (%) for categorical variables and mean ± SD, for continuous variables.

^a^ P-values were calculated using χ2 test or Fisher’s exact test for categorical variables, and Student’s t-test for continuous variables.

Abbreviations: *Helicobacter pylori*, *Helicobacter pylori*.

### Logistic regression analysis of *Helicobacter pylori* recurrence

To further identify risk factors associated with *H. pylori* recurrence, univariate and multivariate logistic regression analyses were conducted (Table 3). In the univariate analysis, three variables were significantly associated with recurrence: prior *H. pylori* treatment failure (P = 0.023), occurrence of antibiotic-associated diarrhea during treatment (P = 0.009), and absence of probiotic use (P = 0.021).

**TABLE 3:** Univariate and multivariate logistic regression analysis of risk factors associated with *Helicobacter pylori* recurrence.

Variable	Univariate OR (95% CI)	P-value	Multivariate OR (95% CI)	P-value
Age ≥50 years	1.28 (0.71–2.32)	0.408	–	–
Male sex	1.14 (0.64–2.04)	0.657	–	–
BMI ≥25 kg/m^2^	0.93 (0.48–1.79)	0.826	–	–
Smoking history	1.32 (0.73–2.41)	0.355	–	–
Alcohol consumption	1.19 (0.65–2.19)	0.572	–	–
Pickled food intake ≥3×/week	1.33 (0.73–2.45)	0.347	–	–
Spicy food intake ≥3×/week	1.24 (0.68–2.27)	0.485	–	–
Family history of gastric cancer	1.66 (0.58–4.70)	0.349	–	–
Chronic gastritis (endoscopic confirmed)	1.17 (0.66–2.06)	0.593	–	–
Peptic ulcer history	1.41 (0.78–2.54)	0.252	–	–
Prior *H. pylori* treatment failure	1.89 (1.06–3.39)	0.031*	1.74 (0.97–3.11)	0.064
Antibiotic-associated diarrhea	2.16 (1.12–4.19)	0.022*	2.04 (1.05–4.00)	0.035*
Bloating during therapy	1.78 (0.91–3.50)	0.092	1.64 (0.83–3.26)	0.154
Incomplete therapy (<90% adherence)	2.37 (1.21–4.61)	0.012*	2.09 (1.06–4.13)	0.033*
Gastrointestinal symptoms at baseline	1.41 (0.77–2.58)	0.261	–	–
High baseline UBT delta (≥20‰)	1.06 (0.60–1.88)	0.839	–	–
Self-reported stress (Likert >7/10)	1.58 (0.88–2.83)	0.123	1.44 (0.77–2.70)	0.252
Family support perceived inadequate	1.31 (0.73–2.37)	0.365	–	–
Probiotic supplementation (yes)	0.47 (0.24–0.91)	0.026*	0.49 (0.25–0.96)	0.037*

Abbreviations: OR: odds ratio; *Helicobacter pylori*, *Helicobacter pylori*.

In the multivariate model, probiotic supplementation emerged as an independent protective factor, associated with a significantly reduced risk of recurrence (OR = 0.49, 95% CI: 0.25–0.96, P = 0.037). Conversely, antibiotic-associated diarrhea was identified as an independent risk factor (OR = 2.04, 95% CI: 1.05–4.00, P = 0.035). These findings reinforce the potential mechanistic relationship between probiotics, gut microbial balance, treatment tolerability, and eradication durability.

## Prognostic outcomes between groups

As shown in Table 4, the probiotic group had a lower rate of unplanned hospital revisits (2.5% vs. 7.5%) and 30-day reconsultations for symptoms (3.3% vs. 8.3%) compared to the control group, though these differences were not statistically significant. However, therapy discontinuation was significantly less frequent in the probiotic group (1.7% vs. 6.7%, P = 0.040), indicating better treatment adherence and tolerability.

**TABLE 4:** Comparison of prognostic outcomes between probiotic and control groups after treatment.

Outcome	Probiotic group (n = 120)	Control group (n = 120)	P-value^a^
*H. pylori* recurrence	11 (9.2%)	23 (19.2%)	0.021
30-day reconsultation for symptoms	4 (3.3%)	10 (8.3%)	0.084
Unplanned hospital revisit	3 (2.5%)	9 (7.5%)	0.069
Therapy discontinuation	2 (1.7%)	8 (6.7%)	0.0

Values are presented as n (%) unless otherwise specified.

^a^ P-values were calculated using χ2 or Fisher’s exact test as appropriate.

^b^ Statistically significant (P < 0.05).

## Discussion

*Helicobacter pylori* remains one of the most prevalent and persistent bacterial infections worldwide, with well-established links to peptic ulcer disease, gastric mucosa-associated lymphoid tissue (MALT) lymphoma, and gastric cancer (Gong and Yuan, 2018; Hanafiah and Lopes, 2020; Matsuo et al., 2017; Sabbagh et al., 2019). While standard quadruple therapy is effective for initial eradication, recurrence remains a significant concern, particularly in regions with high reinfection rates, poor treatment adherence, and widespread antibiotic resistance. This study assessed the impact of probiotic supplementation on recurrence rates and treatment-related outcomes, and our findings suggest that probiotics are associated with reduced recurrence and improved treatment tolerance.

Our results demonstrated that patients who received probiotic supplementation during and after quadruple therapy had a significantly lower recurrence rate of *H. pylori* infection within 12 months compared to those receiving standard care alone. These findings are consistent with prior research indicating that probiotics help restore gut microbiota balance, prevent recolonization by pathogenic organisms, and modulate mucosal immunity (Guo et al., 2023; Luo et al., 2024; Quigley, 2019; Bock et al., 2024). In particular, strains of *Lactobacillus* and Bifidobacterium have shown the ability to inhibit *H. pylori* adherence, reduce inflammation, and promote gastric mucosal healing (Messaoudi et al., 2011).

Furthermore, probiotic use was associated with a reduction in gastrointestinal side effects such as bloating and antibiotic-associated diarrhea—two of the most common reasons for poor adherence and premature treatment discontinuation. Our data also showed that patients in the probiotic group had significantly higher medication adherence rates, which is critical for ensuring successful eradication and preventing antibiotic resistance. These findings are in agreement with meta-analyses by Paoluzi et al. and Kingkaew et al., which concluded that probiotics enhance tolerance and adherence to *H. pylori* eradication regimens (Kingkaew et al., 2023; Paoluzi et al., 2015).

In addition to their immunomodulatory and microbiota-restoring properties, probiotics may also exert indirect benefits by improving patient-reported quality of life during and after eradication therapy. Several studies have shown that gastrointestinal discomfort—particularly bloating, abdominal distension, and altered bowel habits—can significantly impair daily functioning and reduce willingness to complete therapy (Neyrinck et al., 2021; Wood, 2007). By alleviating these symptoms, probiotics not only promote treatment adherence but may also enhance patient satisfaction and long-term health behavior. These patient-centered benefits are increasingly recognized as important indicators of therapeutic value, especially in the context of outpatient care and self-administered regimens. Given the growing interest in microbiome-based interventions, our findings support the integration of probiotics into standard *H. pylori* management protocols as part of a broader, personalized care model.

Importantly, logistic regression analysis confirmed probiotic supplementation as an independent protective factor against *H. pylori* recurrence. Additionally, receiver operating characteristic (ROC) analysis indicated that probiotic use is moderately associated with lower recurrence risk, improved adherence, and reduced adverse events—further supporting its clinical utility as an adjunct therapy.

Despite these encouraging results, several limitations must be acknowledged. First, the retrospective design inherently limits the ability to infer causality. Although propensity score matching was used to balance observed baseline characteristics, the potential for unmeasured or residual confounding cannot be excluded. Therefore, the findings of this study should be interpreted as observational and exploratory in nature. Future prospective, multicenter randomized controlled trials are necessary to validate the causal role of probiotic supplementation in reducing *H. pylori* recurrence. Second, probiotic strains and dosages were not standardized across all patients, which may introduce heterogeneity in treatment effects. Third, recurrence was only monitored for 12 months, and long-term reinfection rates or resistance patterns were not evaluated. Additionally, the study was conducted at a single center, which may limit generalizability.

Future multicenter, prospective randomized controlled trials with longer follow-up durations are warranted to validate our findings, determine optimal probiotic formulations, and explore cost-effectiveness. Investigating the role of specific probiotic strains and duration of administration in different populations could further inform precision-based adjunctive strategies for *H. pylori* eradication.

## Conclusion

In conclusion, this study provides real-world evidence that probiotic supplementation during and after standard quadruple therapy is associated with lower *Helicobacter pylori* recurrence rates, fewer gastrointestinal side effects, and higher treatment adherence. These findings support the incorporation of probiotics as a complementary strategy to enhance eradication durability and improve patient tolerance. Structured probiotic use may represent a simple, safe, and effective intervention to optimize *H. pylori* management in clinical practice. Further high-quality prospective studies are needed to confirm these results and define best-practice guidelines for probiotic integration into eradication protocols.

## Data Availability

The raw data supporting the conclusions of this article will be made available by the authors, without undue reservation.
