# Supplementary material for: The role of probiotic supplementation in reducing Helicobacter pylori recurrence after classic quadruple therapy
Source: Front Pharmacol. 2025 Jul 14;16:1621090. doi: 10.3389/fphar.2025.1621090 (PMC12301312; doi:10.3389/fphar.2025.1621090)
Supplement: Supplementary file 1 [file Table1.docx]

Supplementary Table 1. Subgroup Analysis of Clinical Outcomes Between Probiotic Brand A and Brand B

| Variable | Brand A (n = 58) | Brand B (n = 62) | P-value |
| --- | --- | --- | --- |
| Number of patients | 58 | 62 | — |
| Recurrence rate | 5 (8.6%) | 6 (9.7%) | 0.812 |
| Antibiotic-associated diarrhea | 3 (5.2%) | 3 (4.8%) | 0.943 |
| Bloating | 6 (10.3%) | 7 (11.3%) | 0.861 |
| Treatment adherence ≥90% | 53 (91.4%) | 57 (91.9%) | 0.922 |

Note: Values are presented as n (%). P-values were calculated using chi-square or Fisher's exact test.
